# Lessons learnt from pilot field test of a comprehensive advocacy program to support health promoting schools’ project in Iran

**DOI:** 10.15171/hpp.2017.04

**Published:** 2016-12-18

**Authors:** Towhid Babazadeh, Behrouz Fathi, Abdolreza Shaghaghi, Hamid Allahverdipour

**Affiliations:** ^1^Student Research Committee, Department of Health Education & Promotion, Tabriz University of Medical Sciences, Tabriz, Iran; ^2^Department of Health Education & Promotion, Tabriz University of Medical Sciences, Tabriz, Iran; ^3^Department of Health Education & Promotion, Medical Education Research Center, Tabriz University of Medical Sciences, Tabriz, Iran; ^4^Department of Health Education & Promotion, Clinical Psychiatry Research Center, Tabriz University of Medical Sciences, Tabriz, Iran

**Keywords:** Advocacy, Health promoting schools, School health

## Abstract

**Background: ** Health promoting schools (HPS) project is currently being used in Iran but many challenges still lie ahead. The present study aimed, to test feasibility of implementing a comprehensive advocacy program (CAP) to overcome the obstacles and problems associated with the consummation of school health programs based on the HPS framework.

**Methods:** This quasi-experimental study was performed through recruiting all schools that were enrolled in the national HPS program and located in Jolfa as the intervention group and all of the schools situated in the East Azerbaijan province as control. In order to collect data, Iranian Ministry of Health’s checklists and external audit guidelines for HPS were utilized. In addition, to plan a CAP required data for coordinating fund raising activities including current rules and regulations regarding implementation of local health promotion interventions were collected.

**Results: ** Findings of the study revealed that the implemented CAP had improved all HPS dimensions’ mean score in the intervention group. The observed change in the pre- and post-test score in the intervention group was significant (P < 0.05). The intervention was also successful in encouraging signing of an agreement among the participated organizations to be committed to pursue the adopted policies in facilitation of progress towards full implementation of the local HPS project.

**Conclusion:** Commencing changes in the recruited schools’ structure through coordinated multi-level activities is feasible and this must be considered as a priority where contextual determinants exist to motivate progress towards providing healthier educational settings for school aged children in Iran.

## Introduction

Role of schools’ environments in promoting children’s health is emphasized in many international policy mandates since taking any step in this direction is considered as a direct investment to have healthier next generations. Health promoting schools (HPS) therefore are communities’ asset for countries’ sustainable development especially when limited resources are allocated for children’s wellbeing in larger societies or families.^1,2^

A health promoting school not only improves school aged children’s health through encouraging healthy behaviors and thus prevention of diseases or high risk practices but it may also improve students’ educational performance and achievements.^1-4^ All these acquirements should be considered along with cost savings that communities may gain by decreasing education dropouts or preventing low educational attainments due to reasons such as illnesses or related disabilities.^3^

Antecedence of required revisions in current schools’ environments even goes beyond the local and national boundaries. Therefore it was incorporated into several international health policies including health for all (HFA).^5^ It is also recommended to be one of the effective strategies in filling health gaps among population subgroups and in a larger scale among rural/urban and developed/less developed regions.^3,6^

The health promoting school initiative was introduced in the Ottawa Charter (1980) and addressed as a proper strategy to promote health and empower communities through executing coordinated activities across all sectors (1995). The objectives could be achievable by encouraging communication and collaboration among community sectors and training for better team work.^7,8^

A health promoting school is by definition a school in which continuous efforts are organized to improve students, schools’ staff and members of communities’ health through composing healthy environments, providing required services and dietary recommendations along with maintaining opportunities to improve physical activities and also receive of required mental health counseling.^9,10^ The initiative targets both health and educational activities through mobilizing resources as a framework for collaborative and outcome based efforts to reach HFA goals in schools and larger communities.

The idea was introduced and supported by the World Health Organization (WHO) in the past decade in many countries of the world^11-13^ including Iran.^12^ However, there are challenges in executing the HPS project in several countries. Mobilization of human resources and facilities that are required for execution of the initiative, attracting inter-sectoral collaborations especially between private and governmental organizations and persuading students’ parents and teachers active participations are among the important ones.^10^ Weak or lack of collaborations among responsible organizations, absence of a baseline structure to direct performed activities in a designated route and insufficiency of funding are amongst the important existed barriers in front of the HPS implementation in Iran. Ambiguity of rules and regulations regarding assessment and monitoring of students and school personnel’s health in addition to the scarcity of the health training programs for students’ parents or school personnel have made the current situation even more complicated. All these obstacles along with shortage of human resources such as health counselors and psychologists and current sub-standard physical environments of a considerable number of schools in Iran represent importance of HPS for Iranian students, their parents, schools’ staff and whole country.^14^

Considering the spectrum and nature of the challenges that need to be overcomed to have HPS in Iran, multidimensional interventions are required in local and national levels. These interventions should be planned for lower level stakeholders e.g. students, families and schools’ staff to higher level stakeholder who might be top level policy makers or managers.

Comprehensive advocacy programs (CAPs) could create the required enthusiasm among all stakeholders to work collaboratively in upgrading existent schools to health promoting ones.

To reach the goal changes in policies and practices will be required. Without policy makers and high, middle and lower level managers’ commitment to overcome current barriers in front of the necessary changes that should be planned in the schools, little success must be expectable.^15,16^

This study was designed and carried out to examine feasibility of implementing a CAP and creating stable changes in the schools recruited within the study location (Jolfa, North West of Iran) and to learn lessons for implementation of the planned advocacy program possibly in a wider area or whole country.

## Materials and Methods

This is a quasi-experimental study which utilized pretest-posttest nonequivalent control group design. The study sought to investigate effectiveness of a CAP in promoting the recruited schools towards HPS from February 2011 to October 2013 in Jolfa, North West of Iran. All schools that were determined by the local authorities and compromised to participate in the national level HPS project (n=26) in Jolfa and those schools that had been determined to participate in the national HPS program and located in Tabriz (the capital city of East Azarbaijan province) (n=1041) were assigned to intervention and control groups respectively.

### Measurement

The study data were collected using the introduced checklists for external audit according to the guidelines recommended for assessment of HPS by the Iranian Ministry of Health and Medical Education (MOHME).^12^ The instrument covers eight dimensions including Health Education (8 items which includes 4 one point questions and 4 two point questions); Health Services (11 items which include 5 two points questions and 6 one point questions); Healthy School Environment (12 items with total points ranging between 0-21); Nutrition Services (6 items with total points ranging between 0-12); Physical Education and Physical Activity (5 items with total points ranging from 0-15); Staff Health and Wellness ( 8 items including 5 one point questions and three 2 point questions) and Counseling, Psychological, and Social Services (8 items with total score that range between 0 and 5). In addition; Family and Community Involvement in the HPS’ programs were asked using 8 items that were 6 one point and 2 two points questions. To collect required data for planning of a CAP in the area, several dimensions like allocated funding, published national rules and regulations that support HPS program and official policy documents both in the national and local levels were scrutinized. Using audit checklists, the organized audit teams consisting of health care centers’ staff who were blind to the objective of the study carried out the primary and final assessment of the studied schools regarding the changes that were assumed to take place following the advocacy program.

### Manipulation

A baseline assessment was carried out to recognize the current problems and challenges in successful implementation of the HPS program using the primary assessment checklists. Findings of this stage were used to plan a CAP considering the recommendations were given for conducting a CAP by Sprechmann and Pelton including two steps and in each step the instruments and work sheets introduced by Sprechmann and Pelton were used in the following order.^14^

*(1) Activities prior to conduction of the comprehensive advocacy program *

Main purpose of this phase was to minimize risk of failure and also maximum use of all potentials to achieve success. To accomplish activities of this stage policy documents at local and national scales were checked, key responsible persons in the local organizations, decision makers and those who have capabilities and chance to lobby these policy makers and other important authorities were identified. The identified individuals were contacted consequently to publicize the idea behind the HPS program. Understanding the dominant policy climate in the organizations, all efforts were made to ensure provision of reliable information by credible individuals that included a short list of priorities required to be focused in the planned CAP.

*(2) Planning for comprehensive advocacy program*

Activities of this phase were conducted in the following four stages: analysis of policies, development of strategies for a CAP, making decision on most suitable strategies and program implementation. The performed activities that had been carried out in every stages of this phase were displayed in Table 1.

**Table 1:** Actions taken in the planning stages of the pilot CAP in Jolfa, Iran

**Planning stages**	**Sub-stages**	**Main findings of the stages/sub-stages**
Analysis of policies	Analysis of policies about HPS in Department of Education in Jolfa	- Lack of the policy support of HPS
	Identifying key agencies and organizations	- Aras Free Zone Organization Management, Education Management in Jolfa, Healthy Deputy of Network Health
	Identifying problems	- Lack of appropriate inter-sectoral and intra-sectoral cooperation - Lack of social participation
	Determining items for policy changes	- Adoption of new policies to support of HPS - Developing support programs to support of HPS
Development of strategies for a CAP	Selecting subjects related to the policies	Adoption of new policies to support of HPS
	Identifying the target audience of the program	- Director of the Aras Free Trade Zone‏ - Chairman of the Health Network - Chairman of the Department of Education
	Regulating the aims of policy making	- The adoption and strengthening of supportive policies in support of HPS
Identifying the potential advocators and opponents	- Opposing organizations not found. - Supporting organizations included Department of Education and City Health Network.
Finalizing the strategy of CAP	Identifying the role of every key figure in CAP	- The role of each of the target audiences on how to strengthen the health promoting schools was determined. For example one of the roles of chairman of education was policymaking in order to support of HPS.
	Describing Key messages for the key audience	- A key message designed to Director of Education was as follows: Health promotion in will be schools to improve educational outcomes, reducing risky behaviors and increase the quality of life in the community and schools. Students have the right that school with have international standards and healthier. Your political support of HPS will be improved implementation of the program and the matching it with international standards.
Implementation of the program	Timing of CAP for interviewing with key figures	At this stage, the method and location of connecting with each of the primary target audience was determined.
	Plan evaluation	- Improvement of HPS indicators - Change and approval of policies

Abbreviations: CAP, comprehensive advocacy program; HPS, health promoting schools.

### Statistical analyses 

To examine effectiveness of the implemented advocacy program the collected pre and post intervention data were compared against the data collected in the provincial level following the implementation of baseline HPS program. Data analysis was performed using the statistical package for the analysis of social science (SPSS) version 16. Mean and standard deviation for quantitative data were estimated and inferential statistics of paired *t* test and independent *t* test were applied for comparison purposes. The test results considered to be significant if *P*<0.05.

## Results

The study results revealed that 26 (25%) out of 104 schools that were located in Jolfa area covered by the provincial HPS program and only 3 (11.53%) of them were located in rural areas. Among the 4463 schools that were listed to be working in East Azerbaijan province, 1041 (23.22%) were included in the provincial HPS program of them, 146 (14%) schools were located at rural areas. In the city of Jolfa; 15(57.69%) of the schools were primary schools, 7 (26.92%) were junior high schools and 4 (15.38%) were senior high schools that decided to be included in the provincial HPS program. In East Azerbaijan province, 557 (83.81%) primary schools, 302 (29.17%) junior high schools and 176 (17%) senior high schools were covered by HPS program.

The obtained data regarding the areas of “general policies” and “effective policies” and also in each of the eight mentioned dimensions of the pilot CAP project in Jolfa were summarized in Tables 2 and 3. The policies that were found to boost the HPS program in Jolfa were (1) Integration of the HPS activities into the current formal duties and responsibilities of the participating organizations (2) Strengthening cooperation with local councils to implement the HPS program in educational settings; and (3) Signing of official cooperation agreements with local municipalities, the Red Crescent Office, the Social Welfare organization and also Aras Free Trade Zone (AFTRAZ) authorities to provide support in implementation of HPS program. The policy changes that had been made in all eight dimensions of the CAP program were presented in Table 3. The most important changes that were achieved following the conduction of the CAP included improved level of coordination among local organizations in implementing HPS in the target schools, most better provision of health care for students at recruited schools, planning and implementation of first aid training courses for teachers, schools’ personnel and students by the Red Crescent Office in the region, attraction of the military forces’ cooperation to help in eliminating environmental health problems in the schools prior to initiation of the new educational year, expansion of green spaces through receiving helps from the Natural Resources Preservation Organization as donation of trees and plants, integrating the periodical medical examination of teachers and schools’ personnel into the national-level family physician program and planning of at least one accredited in service training program about health related topics relevant to schools health for teachers by the MOHME’s local offices in the region.

**Table 2:** Changes made in general policies of the participating organization in favor of HPS within the pilot CAP in Jolfa, Iran

**Agreements in local managerial levels**
Considering HPS as one of the priorities in the annual organizational plan and commitment to do team works regarding food safety in the county governance level.
Integration of the all determined health programs into routine organizational activities to support implementation of the local HPS program.
Commitment to cooperate in implementation of the HPS program through organized efforts of the city and rural councils and also school health section of the local headquarters and offices of the Ministry of Education.
To collaborate with other participating organizations including the city council, the city municipality, the city’s Red Crescent Branch, Aras Trade and Industrial Free Zone and Social Welfare Bureau of Jolfa in pursuing the proposed HPS programs.

Abbreviations: CAP, comprehensive advocacy program; HPS, health promoting schools.

**Table 3:** The originated policy changes in the participating schools and local Ministry of Education offices within the pilot CAP in Jolfa, Iran

**Agreed activities to conduct**
Health Education
Implementation of comprehensive health training program at schools for target groups (students, teachers, instructional staff) with cooperation of all participant organizations in the county.
Explanation of the HPS project to students by teachers in all classes. Preparing and giving instructional materials for all students on CDs within the participating schools.
Health Services
Use of surplus human resources working in the district health network as schools’ health officers.
Implementation of first aid in service training courses for all teachers and students in the recruited schools by the local Red Crescent office
Preparing a school health profile for all participant schools in the region with the help of health care staff in the rural or urban health centers
Healthy School Environment
Use of volunteer brigades (Basij members) to improve schools’ environmental health prior to opening of the schools in upcoming educational year
Collaboration with the city Municipality and local office of the Environmental Protection Department to increase green space and donating plants to the schools
Improving safety transportation standards using signs and tags in the streets leading to or around the schools
Nutrition Services
To make having health certificate mandatory for those schools’ staff who provide food to students.
Encouraging healthy diet as an organizational culture (banning distribution of junk foods and unhealthy snacks at the schools, offering healthy foods, fruits …
Staff Health and Wellness
Integration of medical examinations of teachers in the national family physician program and issuing health certificates for them
Planning and implementation of at least one in service training courses related to the schools’ health annually with the cooperation of the local Ministry of Education office
Physical Activity and physical Education
Implementation of physical exercise programs by sport and physical exercises experts.
To give at least a 20 minutes exercise break once a week to teachers at the schools
Counseling, Psychological and Social Services
Implementation of mental health and counseling sessions for students in the schools by the registered mental health experts who work for the Ministry of Education
Identification of the students subjected to risks of social harms and trauma and referring them to appropriate health care service provider e.g. counselors, Social Welfare Bureau or health centers
Making mandatory having of a healthy parenting certificate by parents that indicate their attendance in the related courses to enroll their children in the primary schools
Family and Community Involvement
Approve of the agenda to reinforce collaborations between parents’ councils in the recruited schools and community level organizations or authorities.
Preparing an action plan to integrate health education programs and the routine parents’ education programs within the participant schools
Considering at least one item related to the students’ health in the agenda of every parents council meetings.
Reinforcing peer education at schools
Planning and implementation of briefing sessions about the HPS program in the scheduled list of the activities for parents council meetings

Abbreviations: CAP, comprehensive advocacy program; HPS, health promoting schools.

Effectiveness evaluation of the implemented pilot CAP in Jolfa indicated a significant improvement in the average score of all HPS’ dimensions including health education, physical education and activity, health services, healthy eating, healthy school environment, staff health and wellness, family and community involvement and psychosocial counseling services within the intervention group compared to the control group (*P*<0.001) (Table 4). Additionally, comparison based on the achieved stars according to the five-star rating mechanism showed that the number of schools that were eligible to be appointed at least three stars increased from 3 (13.64%) to 26 (100%) schools after the intervention.

## Discussion

Findings of this pilot field test indicated feasibility of implementing an effective CAP to pursue the proposed polices with regard to execution and development of the HPS project in Jolfa, Iran. The study results were also revealed that (Table 2) the agreements and decisions that have been made following the planning and implementation of the CAP as the main policies to facilitate inter and intra sector collaborations could successfully attract commitment and participation of all stakeholders. Consistent with the findings of this study, several research evidences exist that addresses the importance and efficiency of public participation as one of the essential elements of community involvement in promoting the health programs.^17,18^ Multi-level involvement of stakeholders including families, schools, policy makers and health care providers could increase chance of success to reach the program objectives^16^ and should be regarded as a pivotal principle in implementation of a CAP. Based on the community participatory project for development of HPS in Zhejiang province, China,^17^ considerable improvements in knowledge and behavior of students, staffs, and parents about healthy nutrition were observed. Huang et al also reported that integration of local resources and close cooperation among all collaborators within local communities could greatly help development of HPSs and overcoming challenges stemmed from incongruous activities.^18^ It seems, a participatory approach to HPS would be more than the improvement of the health and would have multidimensional outcomes beyond the schools’ environments.

**Table 4:** Pre and post intervention mean scores of HPS dimensions in the field tested and control schools within the pilot CAP in Jolfa, Iran

**Variables**	**Groups**	**Before intervention**	**After intervention**	***P*** **value** ^b^
		**Mean (SD)**	**Mean (SD)**	
Health Education	Intervention	4.94 (2.26)	9.02 (1.73)	<0.001
	Control	6.24 (3.77)	7.28 (3.52)	0.130
	*P* value^a^	0.150	0.032	
Health Services	Intervention	10.54 (2.20)	12.28 (268)	<0.001
	Control	12.02 (3.86)	12.55 (2.68)	0.404
	*P* value^a^	0.091	0.71	
Healthy School Environment	Intervention	15.69 (2.15)	17.12 (2.16)	0.002
	Control	16.55 (3.71)	16.99 (3.55)	0.512
	*P* value^a^	0.306	0.87	
Nutrition Services	Intervention	5.26 (2.97)	8.57 (1.81)	<0.001
	Control	5.66 (3.81)	6.16 (3.70)	0.472
	*P* value^a^	0.673	0.002	
Physical Education and Physical Activity	Intervention	4.09 (0.92)	4.84 (0.33)	<0.001
	Control	4.16 (1.14)	4.27 (1.08)	0.592
	*P* value^a^	0.807	0.010	
Staff Health and Wellness	Intervention	3.27 (2.26)	6.53 (1.39)	<0.001
	Control	3.18 (2.58)	3.79 (2.62)	0.209
	*P* value^a^	0.900	0.001	
Counseling, Psychological, and Social Services	Intervention	3.42 (2.51)	6.96 (1.19)	<0.001
	Control	7.02 (3.26)	7.49 (3.01)	0.419
	*P* value^a^	0.000	0.403	
Family and Community Involvement	Intervention	3.50 (2.59)	6.30 (2.31)	<0.001
	Control	5.48 (3.09)	5.97 (2.97)	0.383
	*P* value^a^	0.012	0.654	

Abbreviations: CAP, comprehensive advocacy program; HPS, health promoting schools. ^a^Independent *t* test; ^b^Paired *t* test.

Results of other studies were also indicative of the effectiveness of CAPs in improving and sustaining health programs.^18-20^ Winkleby et al, based on implementation of a CAP, have reported a considerable decrease in cigarettes consumption in their study’s intervention group.^19^ Goodkind, reported that through a CAP, satisfaction of the Hmong refugees about assessment process of their required resources, living standards and stress level had improved.^20^

While the conducted CAP had considerable outcomes for development of HPS project in the study location, but various challenges were also encountered including lack of full cooperation of responsible organizations that interfered with expected achievements. Failure in achieving full cooperation of all stakeholders in the HPS programs were also reported in other studies.^1,21-25^ In spite of the importance of inter-sectoral collaboration in success of community wide programs, the required level of cohesion that is especially required in developing countries was not seen due to partially rigid and patchy definition of organizational responsibilities and priorities.^26^

Implementation of CAP may face same common route barriers and challenges in the developed and developing countries^27^ with potential to influence the program results. In this study, the principal encountered challenges in implementation of CAD program were as follows: instability of directors and managers in their position in the relevant organizations, resisting against the required changes in the organizations’ responsibilities, higher priority of the short term benefits compared to long term organizational achievements, lack of culture of team work and full cooperation to achieve organizational goals and objectives and deficiencies in financial resources that are needed for coordinated activities. Consistent with the findings of this study, Abdulmalik et al^27^ have pointed to the facing challenges in implementation of CAP such as lack of full support from the policy makers side due to a narrow level of expertise they may have and reluctance of official staff to input new activities or objectives into their organizational routines that may expand their current responsibilities beyond the existent frameworks.^27^

## Limitations

While there is no explicit blueprint to reach success in advocacy programs all efforts were made to use current research evidence in planning phase of the study. An unequivocal hybrid of professional skills to fascinate partnership was applied to build local capacity for supporting the initiative. Thus; local planning skills for HPS were improved but we believe that overall capacity to sustain the required support for long term planning remained inconsistent. A part of this paucity was related to bureaucracy of decision makings in the local organizations, hierarchy of the agreements’ approval in the upper level managerial settings and divergent priorities within the participant organizations.

To achieve advocacy programs’ goals proactive collaboration of all stakeholders is indispensable but financial impediments were amongst the most alleged considerations by the local level managers to intimate participation with the scheduled advocacy program. The current top-down centralized planning and budgeting mechanisms in Iran prohibits creativity in local level decision makings and prioritization of the health problems.

Another limitation of this study was abstaining of a number of local stakeholders from full engagement in the designed HPS reinforcement project.

Another limitation of this study was assuming of existent baseline data in provincial level suitable for comparison purposes. This is while; inherent differences actually might exist between the study location and other socio-economic milieus. We were aware of importance of selecting a socio-economically and demographically homogenous control field but due to logistical restraints it was impracticable. Despite all these impediments the study results could provide advices for better planning and implementation of HPS programs in at least socio-culturally and economically similar settings.

## Conclusion

This study used the strategy of CAP as an attempt to overcome the potential challenges and obstacles in front of implementing a local version of the HPS program in a typical national level setting. The study findings indicated that using a well-organized CAP and implementation of a sustainable HPS program could greatly improve the current programs and overcome many challenges. The employed strategies and lessons learnt in this local CAP may help other local or even national level programs in progressing towards healthier schools and thus healthier next generations. While, the identified barriers and applied strategies could be applicable in other national and even international levels, special attention must be paid to the inherent differences that might exist and interfere with the CAP implementation in culturally, socially or organizationally heterogeneous settings.

## Implication for policy and practice

Based on the study findings advocacy activities for boosting national level HPS initiatives should consider more effective routes to engage all organizations and administrative sectors in public health activities as one of their national and even international missions that need to be followed for a safer and promising planet for all. Secondly, ministries of health and relevant organizations should clarify their expectations about how other organizations can engage and collaborate in improvement of school health programs and help them to move in line with health promotion programs in a sustained manner. Next, all organizations are encouraged to have a clear and documented policy to help local and national level health promoting programs to overcome national health related challenges since; weak or lack of inter-sectoral cooperation might hamper accurate implementation of community based health promoting programs.

## Ethical approval

The ethical approval was obtained from the Human Research Ethics Committee (HREC) of the Tabriz University of Medical Sciences. In line with the ethical guidelines for implementation of the study, its purpose was clearly explained to the participants i.e. involved organizations and groups. The participants’ rights to withdraw from the study at any time for any reason and without any obligation in giving reasons for their refusal to continue participation in the study were also clarified. In the implementation phase of the study during negotiations and convincing study participants their views, times and preferred schedules for spending time to answer questions or give their opinions were considered respectfully. All efforts also were made to build mutual trust with the respondents and carefully concede issues like organizational benefits, sensitivity of the managerial tasks and other considerations.

## Competing interests

None of the authors has any conflict of interest.

## Authors’ contributions

TB helped in drafting the article and data analysis, BF major role was conceptualization and design of the study, data collection and interpretation and drafting the article, ASH and HA helped greatly in conceptualization and design of the study, data analysis and interpretation and revised several drafts of the article critically for major intellectual content. All authors have read and approved the submitted and revised final version of the manuscript.

## Acknowledgments

This research was supported by a grant from the Tabriz University of Medical Sciences. The authors would like to appreciate all local organizations’ support in execution of this study.

## References

[1] Ippolito-Shepherd J, Cerqueira MT, Ortega DP (2005). Health-Promoting Schools Regional Initiative of the Americas. Promot Educ.

[2] St Leger L, Young I, Blanchard C, Perry M. Promoting health in schools: from evidence to action, 2010. 2010 [cited 30 March, 2012]. Available from:http://hivhealthclearinghouse.unesco.org/library/documents/promoting-health-schools-evidence-action.

[3] WHO Office of Public Information Geneva. WHO’S Global school health initiative: helping schools to become Health Promoting Schools. Geneva: WHO; 1998 [cited 13 May, 2012]. Available from:https://apps.who.int/inf-fs/en/fact092.html.

[4] Lee A, Cheng FF, Fung Y, St Leger L (2006). Can health promoting schools contribute to the better health and wellbeing of young people? The Hong Kong experience. J Epidemiol Community Health.

[5] Fathi B, Allahverdipour H, Shaghaghi A, Kousha A, Jannati A (2014). Challenges in developing health promoting schools’ project: application of global traits in local realm. Health Promot Perspect.

[6] Shama ME, Abdou SS (2009). Evaluating the impact of health promoting school initiative on dietary habits and BMI of students in Oman. J Egypt Public Health Assoc.

[7] World Health Organisation (WHO). Health Promoting Schools: A Healthy Setting for Living, Learning and Working. Geneva: WHO; 1998.

[8] Health-Promoting Schools Promoting the World Health Organisation’s concept of health. Unesco International Science Tecnology & Environmental Education Newsletter 1998;23(2):1-16.

[9] World Health Organization. Improving Health Through Schools: National and International Strategies. Information Series on School Health. Geneva, Switzerland: WHO; 1999.

[10] World Health Organization. What is a health promoting school. Available from: http://www.who.int/school_youth_health/gshi/hps/en/.

[11] St Leger LH (1999). The opportunities and effectiveness of the health promoting primary school in improving child health—a review of the claims and evidence. Health Educ Res.

[12] Motlagh M, Dashti M, Moslemi M, Aminaei T, Ardalan G. Guidline for the implementation of the health promoting schools in Islamic Republic of Iran. 2nd ed. Ghom: Khademoleza; 2011. [Persian].

[13] Mũkoma W, Flisher AJ (2004). Evaluations of health promoting schools: a review of nine studies. Health Promot Int.

[14] Sprechmann S, Pelton E. Advocacy Tools and Guidelines: Promoting Policy Change. Atlanta, GA: CARE; 2001.

[15] World Health Organization. Health Promotion Glossary. Geneva: World Health Organization; 1998.

[16] Ayer V, Bunn C. Advocacy Expert Series: Book 1 Advocacy Campaign Management. 1st ed. Pact Cambodia; March 2004. Available from: http://www.pactworld.org/galleries/resource-center/advocacy_series_module1.pdf.

[17] Shi-Chang X, Xin-Wei Z, Shui-Yang X, Shu-Ming T, Sen-Hai Y, Aldinger C (2004). Creating health-promoting schools in China with a focus on nutrition. Health Promot Int.

[18] Huang JJ, Yeh GL, Tseng CC, Chen WW, Hwu YJ, Jiang DD (2009). Using organization development concept to conduct administrative assessment of health promoting schools in Taiwan- A Preliminary Study. Int Electron J Health Educ.

[19] Winkleby MA, Feighery E, Dunn M, Kole S, Ahn D, Killen JD (2004). Effects of an advocacy intervention to reduce smoking among teenagers. Arch Pediatr Adolesc Med.

[20] Goodkind JR (2005). Effectiveness of a community-based advocacy and learning program for hmong refugees. Am J Community Psychol.

[21] Davis C, Salo L, Redman S (2001). Evaluating the effectiveness of advocacy training for breast cancer advocates in Australia. Eur J Cancer Care (Engl).

[22] Inchley J, Muldoon J, Currie C (2007). Becoming a health promoting school: evaluating the process of effective implementation in Scotland. Health Promot Int.

[23] Bruce E, Klein R, Keleher H (2012). Parliamentary inquiry into health promoting schools in Victoria: analysis of stakeholder views. J Sch Health.

[24] Barnekow Rasmussen V (2005). The European Network of Health Promoting schools-from to Kyrgyzstan. Promot Educ.

[25] Thomas M, Benton D, Keirle K, Pearsall R (1998). A review of the health promoting status of secondary. Health Promot Int.

[26] Young I, St Leger L, Blanchard C. Monitoring and assessing progress in health promoting schools: issues for policy makers to consider. Saint Denis, France: IUHPE; 2012.

[27] Abdulmalik J, Fadahunsi W, Kola L, Nwefoh E, Minas H, Eaton J (2014). The Mental Health Leadership and Advocacy Program (mhLAP): a pioneering response to the neglect of mental health in Anglophone West Africa. Int J Ment Health Syst.

